# Genome-Wide Superfamily Profiling Reveals Key Regulator *LsAP2/ERF10* That Governs Petal Morphology in *Lagerstroemia speciosa*

**DOI:** 10.3390/plants15162427

**Published:** 2026-08-09

**Authors:** Zhiting Wan, Mao Lin, Yu Huang, Chunmei Yu, Qixiang Zhang, Huitang Pan, Tangchun Zheng

**Affiliations:** 1 Beijing Key Laboratory of Ornamental Plants Germplasm Innovation & Molecular Breeding, National Engineering Research Center for Floriculture, Beijing Laboratory of Urban and Rural Ecological Environment, Engineering Research Center of Landscape Environment of Ministry of Education, Key Laboratory of Genetics and Breeding in Forest Trees and Ornamental Plants of Ministry of Education, School of Landscape Architecture, Beijing Forestry University, Beijing 100083, China; 2 Guangxi Laboratory of Forestry, Guangxi Forestry Research Institute, Nanning 530002, China; 3 College of Art and Design, Nanning University, Nanning 530200, China; 4 School of Life Sciences, Nantong University, Nantong 226019, China

**Keywords:** *Lagerstroemia speciosa*, genome-wide identification, AP2/ERF, floral organ development, expression pattern

## Abstract

Double-flowered cultivars are generally considered more attractive than single-flowered varieties in ornamental plants. The AP2/ERF transcription factors superfamily plays pivotal roles in plant development, including floral organ formation. Here, a total of 248 AP2/ERF genes were identified in the genome of *L. speciosa*, and these genes were unevenly distributed on the 24 chromosomes. Phylogenetic analysis classified LsAP2/ERF genes into five distinct groups; the ERF subfamily was the largest, whereas the AP2 subfamily was associated with floral development. Gene duplication events contributed to the expansion of *LsAP2/ERF* family members, with segmental duplication identified as the primary contributor. Promoter cis-element analysis revealed an abundance of light-responsive and hormone-responsive elements. Spatiotemporal expression profiling showed that core AP2 subfamily members (*LsAP2/ERF4/9/10/13/219*) exhibited distinct expression patterns during flower bud development. Moreover, heterologous overexpression of *LsAP2/ERF10* in tobacco resulted in transgenic lines with altered petal morphology, supporting its potential functional involvement in floral development. This study provides comprehensive characterization of the AP2/ERF family in *L. speciosa*, laying the foundation for elucidating its molecular mechanisms in floral morphogenesis.

## 1. Introduction

Transcription factors (TFs) are functional proteins that specifically recognize and bind to cis-acting elements in promoter regions of eukaryotic genes, playing pivotal roles in transcriptional regulation during plant growth and development [1,2]. Among these, the AP2/ERF family represents one of the largest and most diverse TF families in plants, characterized by at least one AP2 conserved domain composed of 60–70 amino acids [3]. This domain mediates specific binding to cis-regulatory elements, such as GC-boxes (ERF subfamily) and CRT/DRE motifs (DREB subfamily), to regulate adaptation to biotic and abiotic stresses [4,5]. Based on the number and sequence features of AP2/ERF domains, the family is systematically classified into five subfamilies: AP2, ERF, DREB, RAV, and Soloist [5,6]. ERF and DREB proteins generally recognize different core cis-regulatory elements, whereas the DNA-binding specificity may vary among individual family members depending on sequence divergence and functional specialization. Recent advances in genome sequencing have facilitated the identification of AP2/ERF members across diverse plant species. The AP2/ERF family exhibits varying numbers due to genome size differences in different species, including hybrid tea rose [7], *Pennisetum glaucum* [8], *Dimocarpus longan* [9], and *Ananas comosus* [2]. These studies have not only expanded our understanding of AP2/ERF TFs but also highlighted their essential roles in plant development [10,11].

In floral development, AP2/ERF family members, particularly the AP2 subfamily, have been demonstrated to regulate organ identity, flowering time, and floral morphogenesis [12,13]. The ABCDE model identifies *APETALA2* (*AP2*) as a class A gene essential for sepal and petal development [14,15]. *AP2* disruption causes petal-to-stamen homeotic transformation, while its miR172-binding site mutation leads to enlarged meristems and increased petal number [16,17,18]. In *A. thaliana*, AP2 and its homologs not only participate in floral meristem establishment but also act as central regulators of sepal and petal identity by modulating downstream floral development genes [19]. Beyond the AP2 subfamily, recent studies revealed that ERF members (such as *RhERF1/4*) coordinate ethylene and auxin signaling pathways to regulate pectin metabolism, thereby influencing petal senescence and abscission [20]. The RAV subfamily member *TEM1* suppresses flowering by binding to the 5′ untranslated region (5′UTR) of the florigen gene *Flowering locus T* (*FT*) [21]. Notably, AP2/ERF genes in ornamental species such as *Lycoris* and Orchidaceae control floral morphology and pigmentation, making them prime targets for molecular breeding [22,23].

*Lagerstroemia speciosa*, a deciduous tree native to Southeast Asia, exhibits medicinal and ornamental value [24,25]. As a summer-flowering woody species, it is widely cultivated in landscapes due to its striking floral displays, tall stature, and prolonged blooming period. The double-flowered variety ‘Yunshang’, which originated from a natural bud mutation of *L. speciosa*, exhibits a stamen petalody phenotype, providing a unique genetic resource for studying double-flower traits in woody ornamentals [26,27]. Although a chromosome-level genome assembly of *L. speciosa* has recently been released, which laid a solid molecular basis for gene functional research [28], most functional characterizations of AP2/ERF genes have been conducted in model plants and several crop species, and their roles in regulating floral morphological diversity in woody ornamental plants remain largely unexplored. In particular, the composition of the AP2/ERF family in *L. speciosa*, the evolutionary mechanisms underlying its expansion, and the expression divergence of *AP2/ERF* members between single- and double-flowered materials have not been systematically investigated. Therefore, comprehensive identification and characterization of *AP2/ERF* genes in *L. speciosa* may provide new insights into the molecular regulation of floral organ development.

In this study, we systematically identified 248 *AP2/ERF* family members. Evolutionary characteristics were elucidated through bioinformatics analyses including phylogenetic analysis, chromosomal localization analysis, cis-acting element prediction and duplication event analysis. By integrating expression profiling with functional validation, we screened and identified candidate regulators contributing to petal morphogenesis. *LsAP2/ERF* genes exhibited spatiotemporal expression differences between single- and double-flowered floral buds, suggesting that some members may play roles in petal development. These findings provide novel insights into the molecular mechanisms underlying AP2/ERF-governed floral organogenesis in woody plants while refining molecular design breeding strategies for ornamental species, especially for the synergistic improvement of complex floral architecture.

## 2. Results

### 2.1. Genome-Wide Identification of AP2/ERF Genes

To systematically identify AP2/ERF family members in *L. speciosa*, genome-wide searches were performed using conserved AP2/ERF domain sequences from *A. thaliana* as queries. After removing redundant sequences and confirming the presence of conserved AP2/ERF DNA-binding domains, a total of 248 *LsAP2/ERF* genes were identified in the *L. speciosa* genome. Among all AP2/ERF proteins, the shortest protein was 102 aa (LsAP2/ERF241), while the longest was 740 aa (LsAP2/ERF171). The molecular weights of *L. speciosa* AP2/ERF proteins ranged from 11.82 kDa (LsAP2/ERF241) to 79.46 kDa (LsAP2/ERF171), with predicted isoelectric points (pI) ranging from 4.48 (LsAP2/ERF168) to 10.43 (LsAP2/ERF56). Detailed information for all 248 AP2/ERF genes is provided in Table S1.

### 2.2. Phylogenetic Analysis and Classification of AP2/ERF Genes

**Figure 1:**
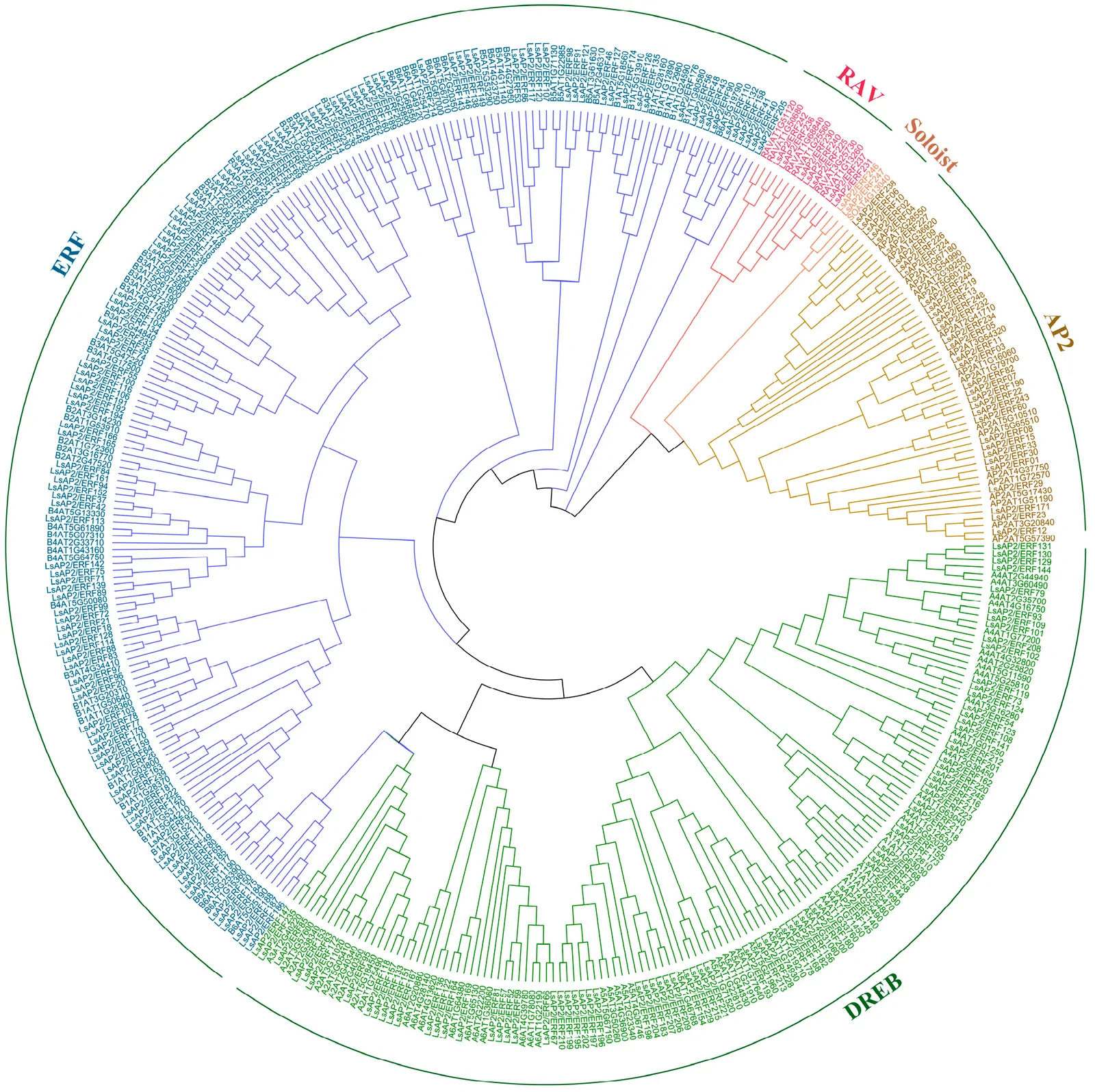
Phylogenetic trees of AP2/ERF genes in *L. speciosa* and *A. thaliana*.

To elucidate phylogenetic relationships among AP2/ERF superfamily proteins in *L. speciosa* and establish a more refined classification, the full-length AP2/ERF protein sequences from *L. speciosa* and *A. thaliana* were aligned for phylogenetic analysis, and a comprehensive phylogenetic tree of AP2/ERF family proteins was constructed for detailed classification (Figure 1). Based on phylogenetic relationships, LsAP2/ERF proteins were grouped into five groups, corresponding to AP2, ERF, DREB, RAV and Soloist. The ERF subfamily contained the largest number of genes (124), followed by the DREB subfamily with 82 genes. The RAV subfamily contained 7 genes. The Soloist subfamily, being the smallest group (comprising 3 genes), exhibited closer evolutionary affinity to AP2 than to DREB. Phylogenetic analysis revealed clear divergence: all AP2 genes formed a distinct clade separate from ERF genes, with an evident boundary between DREB and ERF subfamilies. Notably, the AP2 subfamily, which is associated with floral development, contained 32 genes.

### 2.3. The Cis-Elements in the Promoters of AP2/ERF Genes

**Figure 2:**
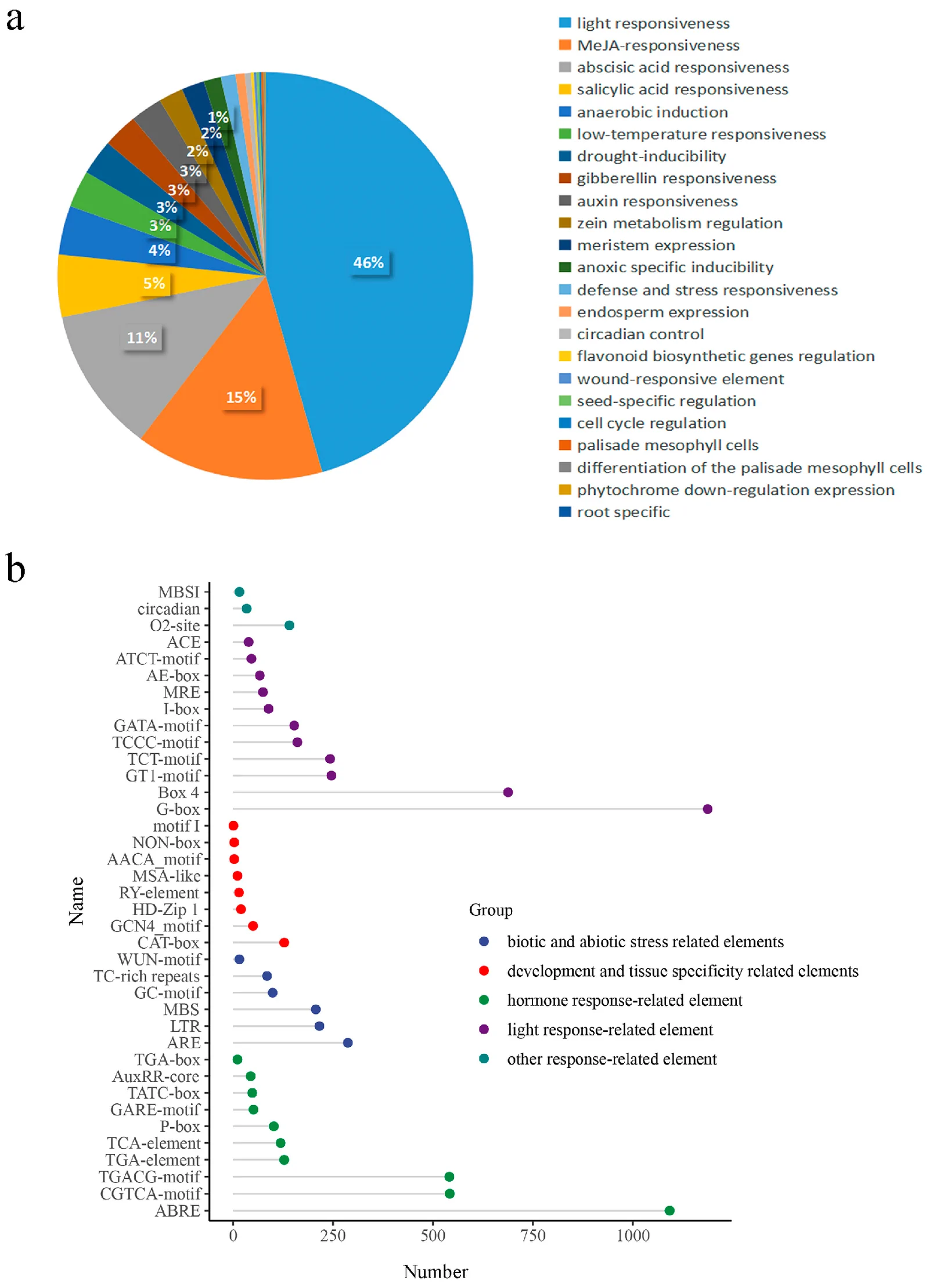
Promoter cis-acting element analysis of *LsAP2/ERF* family genes. (**a**) The proportion of cis-elements predicted in the promoters of *AP2/ERFs*; Note: the legend on the right lists all cis-element types from top to bottom in decreasing order of frequency, while the pie chart shows the percentage values for elements exceeding 1%; (**b**) number of cis-elements involved in biotic and abiotic response, tissue specificity, hormone response, and light response functions and other special functional elements.

To investigate the potential functions of the AP2/ERF gene family in *L. speciosa*, we performed a systematic analysis of cis-acting elements in the promoter regions of *LsAP2/ERF* genes. The results demonstrated that the promoter regions of *LsAP2/ERF* genes contain an abundance of regulatory elements related to plant growth and development, hormone responses, and abiotic stress responses (Figure 2a). A total of 23 functional categories of elements were identified, comprising 5 types of hormone-responsive elements, 6 types of stress-related elements, 7 types of developmental regulatory elements, and 5 types of special functional elements. Notably, light-responsive elements were ubiquitously distributed in the majority of *LsAP2/ERF* gene promoters, suggesting this gene family’s potential involvement in light-mediated developmental regulation.

Regarding hormone responses, in addition to ABREs, the promoter regions were enriched with several characteristic elements: CGTCA motifs and TGACG motifs (MeJA-responsive elements), TCA elements (salicylic acid responsiveness), GARE motif/P-box/TATC-box (gibberellin responsiveness), and AuxRR-core/TGA elements (auxin responsiveness). These findings indicate that *LsAP2/ERFs* may participate in plant physiological processes through a coordinated multi-hormone regulatory network. The stress-responsive elements exhibited remarkable diversity, including LTRs (low-temperature responsiveness), MBSs (drought inducibility), GC motifs (anoxia-responsive), and TC-rich repeats (defense and stress responsiveness). Among the developmental regulatory elements, CAT-box elements associated with meristem activity were detected in 94 gene promoters, endosperm-specific expression elements in 21 gene promoters, and root-specific regulatory elements in 15 gene promoters. Particularly noteworthy was the specific distribution of palisade mesophyll cell differentiation-related elements in 12 genes, implying this gene family’s potential role in leaf morphogenesis regulation (Figure 2b).

### 2.4. Chromosomal Distribution and Genomic Duplication of AP2/ERF Genes

**Figure 3:**
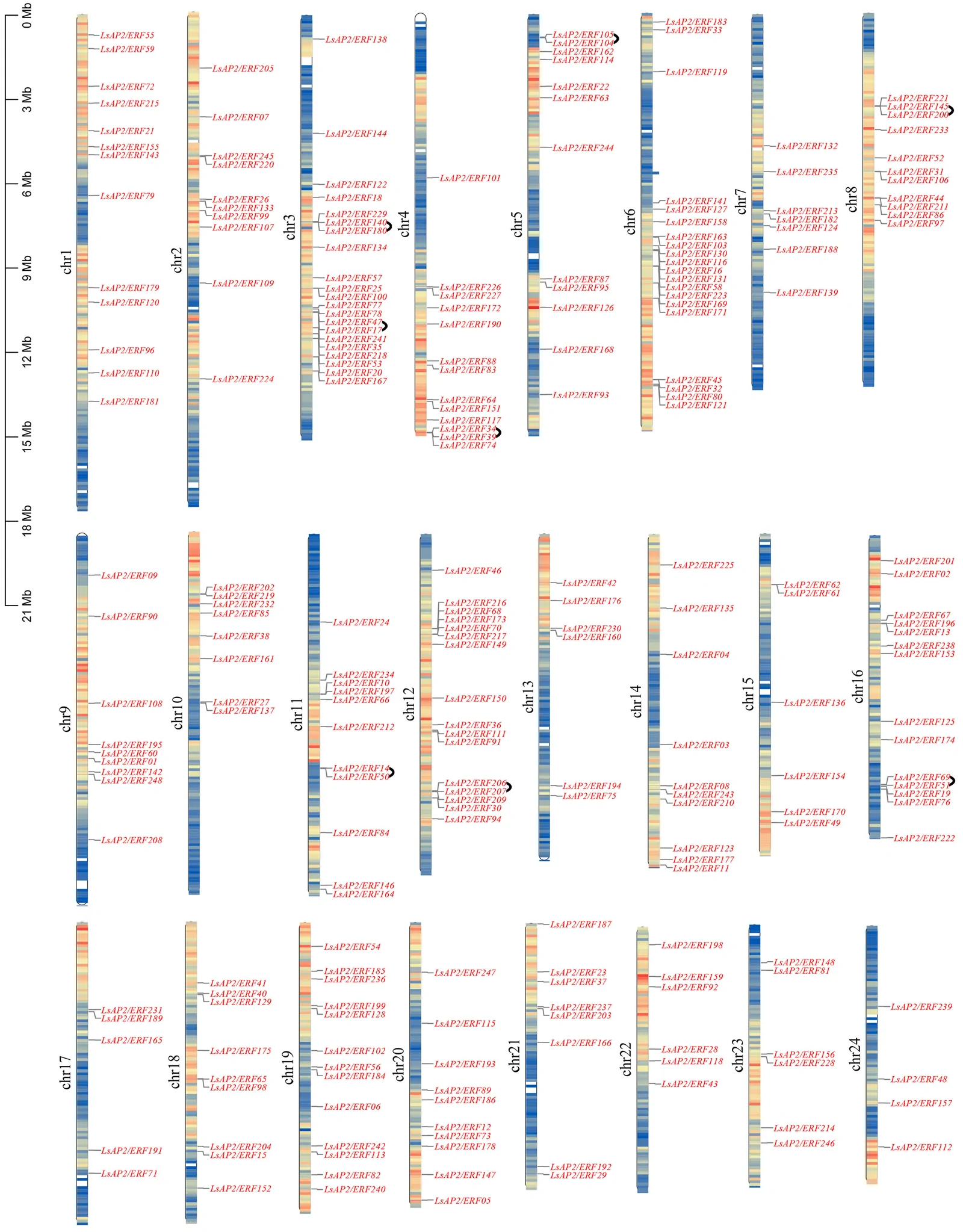
Distribution of 248 *LsAP2/ERF* genes on chromosomes.

**Figure 4:**
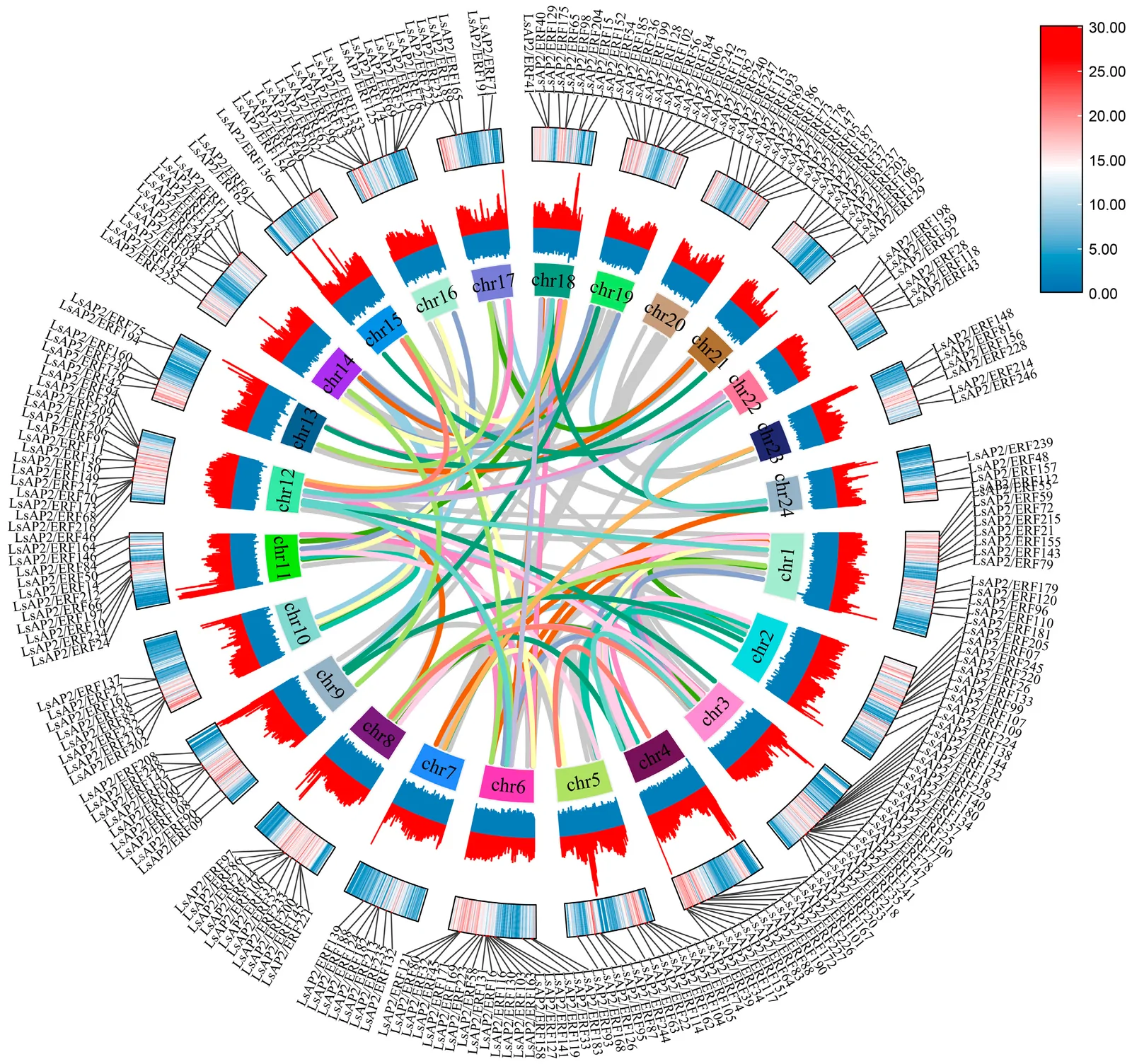
Segmental duplication of *LsAP2/ERF* genes in 24 chromosomes. The tracks from outside to inside are: gene density, GC content, distribution of unknown bases, 24 chromosomes length and syntenic blocks.

To investigate the duplication events of *LsAP2/ERF* genes in *L. speciosa*, we conducted tandem and segmental duplication analyses (Figure 3). The 248 *LsAP2/ERF* genes were unevenly distributed across 24 chromosomes in *L. speciosa*. We identified eight pairwise tandemly duplicated gene relationships (16 genes in total), which were distributed on Chr3, Chr4, Chr5, Chr8, Chr11, Chr12, and Chr16 (Figure 3). These tandem duplication genes include members of the DREB subfamily, with 3 pairs (*LsAP2/ERF140-LsAP2/ERF180*, *LsAP2/ERF145-LsAP2/ERF200*, *LsAP2/ERF206-LsAP2/ERF207*), and members of the ERF subfamily, with 5 pairs (*LsAP2/ERF17-LsAP2/ERF47*, *LsAP2/ERF34-LsAP2/ERF39*, *LsAP2/ERF104-LsAP2/ERF105*, *LsAP2/ERF14-LsAP2/ERF50*, *LsAP2/ERF51-LsAP2/ERF69*). Furthermore, we found that the expansion of the *LsAP2/ERF* family primarily originated from segmental duplications. A total of 184 pairs of segmentally duplicated genes were identified on the chromosomes (Figure 4), and these segmentally duplicated gene pairs were mainly concentrated on Chr1 and Chr12.

### 2.5. Syntenic Analysis of AP2/ERF Genes Between L. speciosa and Other Plants

**Figure 5:**
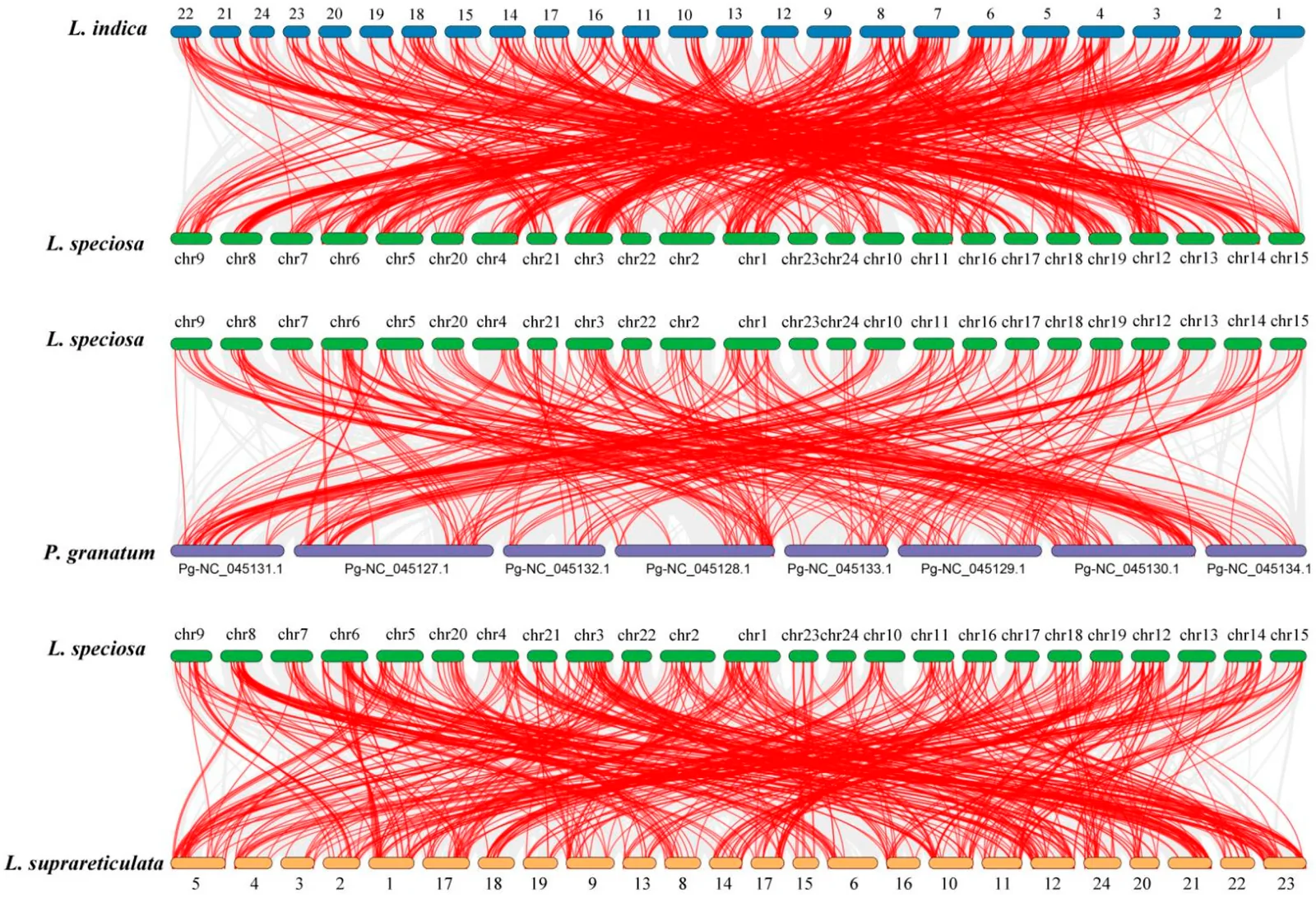
Synteny relationships among homologous *AP2/ERFs* in four representative plant species.

In order to further understand the potential evolutionary clues of *AP2/ERF* genes, we conducted genomic collinearity analysis of representative plants of the Lythraceae, namely *Lagerstroemia indica*, *L. speciosa*, *Lagerstroemia suprareticulata*, and *Punica granatum.* These results revealed significant collinear relationship among *Lagerstroemia* species, notably highlighting that the number of homologous genes in phylogenetically distant *Punica granatum* was smaller than that of congeneric *Lagerstroemia* species. Among them, the highest number of homologous gene pairs was between *L*. *suprareticulata* and *L. speciosa* (Figure 5).

### 2.6. Expression Pattern of AP2/ERF Genes at Different Developmental Stages

**Figure 6:**
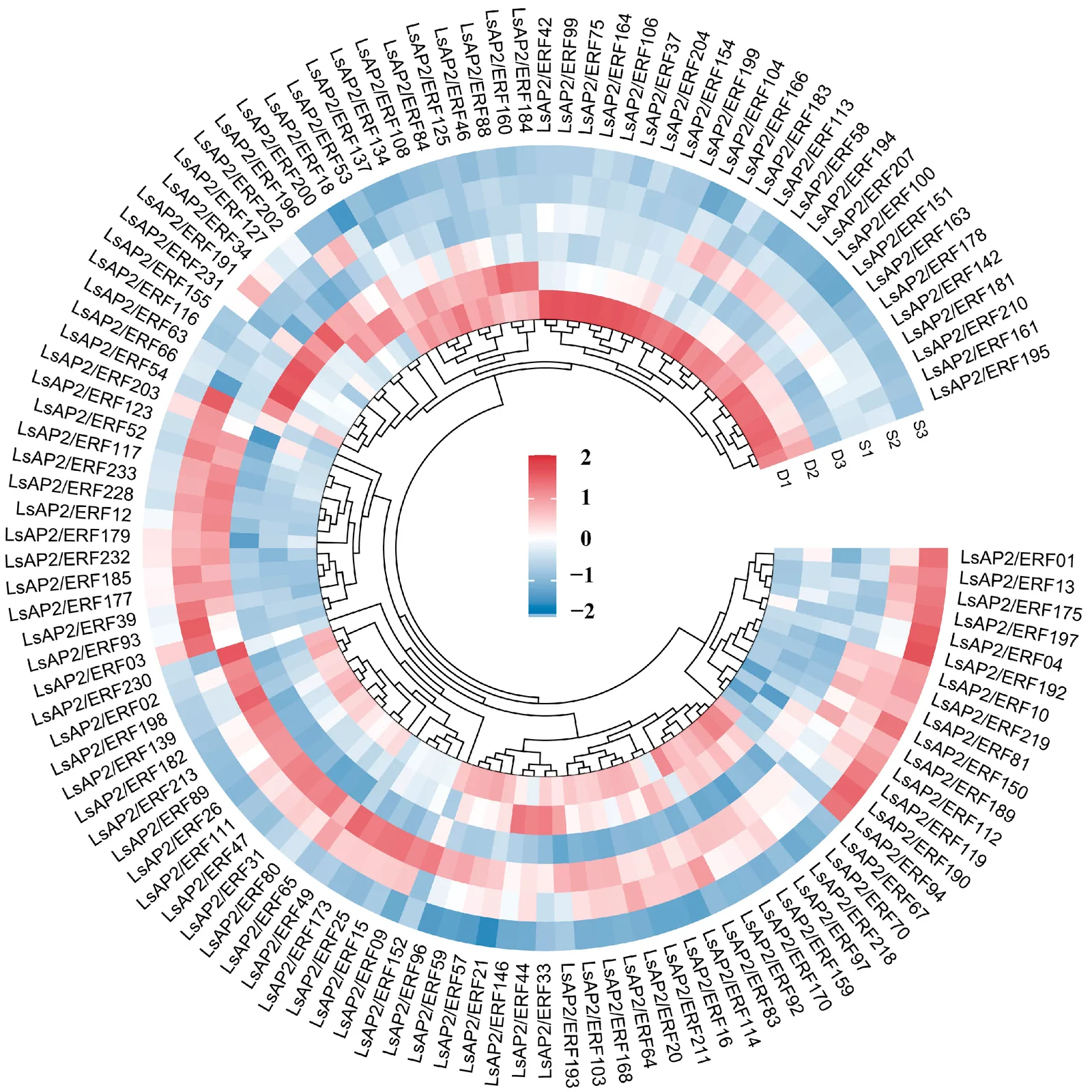
Expression patterns of *LsAP2/ERF* genes in different developmental stages. D1–D3, the first three developmental stages of double-petal bud formation; S1–S3, the first three developmental stages of single-petal bud formation.

Using transcriptome data of single-petal and double-petal flowers at different developmental stages, we detected and analyzed the expression patterns of *LsAP2/ERF* genes during various floral bud developmental phases (Figure 6). Among them, 119 *AP2/ERF* genes were expressed during flower bud development and exhibited differential expression trends across different developmental stages. Most of these genes were expressed during the first two stages of both single-petal and double-petal flower bud development, such as *LsAP2/ERF168* and *LsAP2/ERF170*. In contrast, *LsAP2/ERF12* and *LsAP2/ERF232* were significantly expressed only in the S1 and S2 stages of single-petal flower buds. Notably, compared with double-petal flowers, core AP2 subfamily genes (*LsAP2/ERF1*, *LsAP2/ERF13* and *LsAP2/ERF4*) were predominantly expressed in the later stages of single-petal flower bud, whereas *LsAP2/ERF9* was specifically detected at the early developmental stages of single-petal flower buds. Meanwhile, *LsAP2/ERF10* and *LsAP2/ERF219* were expressed throughout all three developmental stages of single-petal flower buds.

### 2.7. Prediction of the Interaction Network Between AP2/ERF Proteins

**Figure 7:**
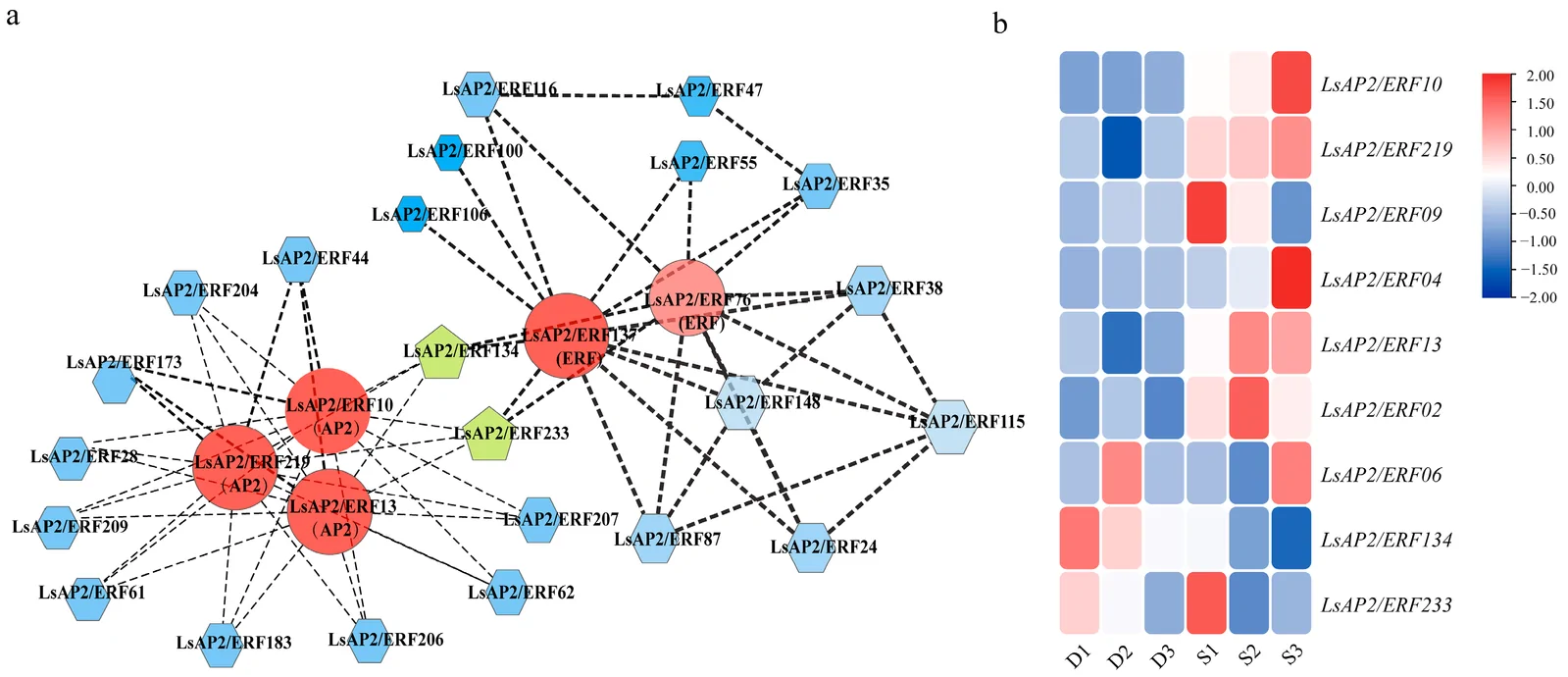
Analysis of potential associations among LsAP2/ERF proteins. (**a**) AP2/ERF protein interaction network diagram; (**b**) qRT-PCR validation of the core genes.

The predicted interaction networks of AP2/ERF proteins in regulating floral development showed that the proteins with relatively high association degrees were LsAP2/ERF10, LsAP2/ERF219, and LsAP2/ERF13, all of which belong to the euAP2 subfamily (Figure 7a). Members of this subfamily were primarily associated with floral development. Proteins predicted to interact with them were mostly ERF subfamily members, including LsAP2/ERF134, LsAP2/ERF233, and LsAP2/ERF76. Additionally, RT-qPCR analysis of the core genes found that the expression patterns of most interacting genes indicated their predominant expression in single-petal plants (Figure 7b). Specifically, *LsAP2/ERF10* exhibited significant expression during the S3 stage, while *LsAP2/ERF9* showed high expression during the S1 stage. In contrast, *LsAP2/ERF219* and *LsAP2/ERF13* were expressed across all developmental stages of single-petal flower buds. These genes displayed differential expression patterns between single-petal and double-petal flowers, suggesting their potential involvement in the bud development process of *L. speciosa*.

### 2.8. Subcellular Localization Analysis of AP2/ERF Proteins

**Figure 8:**
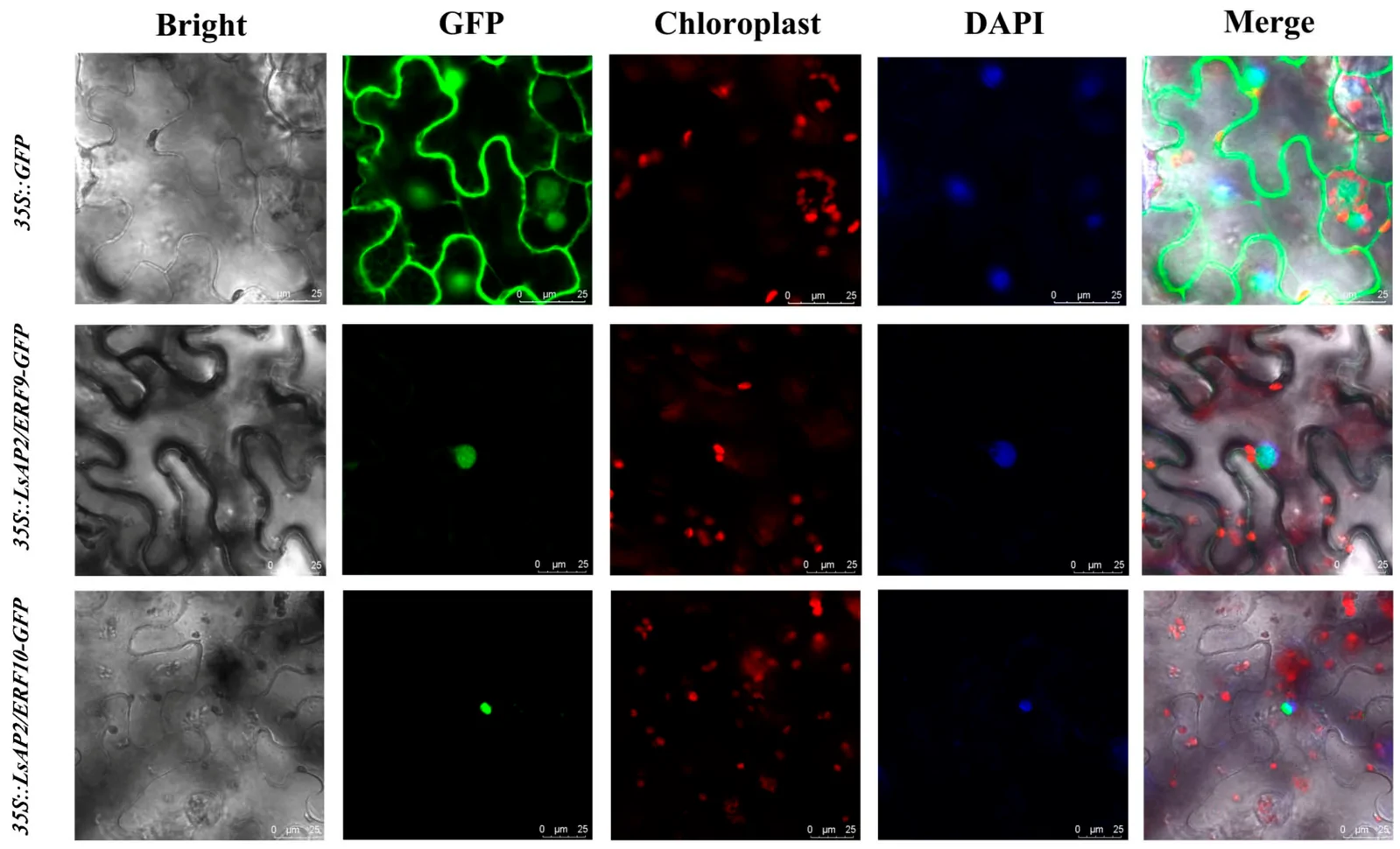
Subcellular localization of LsAP2/ERF proteins in *N. benthamiana* leaves.

To determine the subcellular localization of LsAP2/ERFs proteins, their coding sequences were fused with GFP and transiently expressed in tobacco. Confocal laser scanning microscopy revealed that the proteins encoded by LsAP2/ERF9 and LsAP2/ERF10—two genes specifically expressed in single-petal flower buds—are predominantly localized in the nucleus (Figure 8), aligning with typical characteristic of transcription factors.

### 2.9. Functional Validation of LsAP2/ERF10 Genes

**Figure 9:**
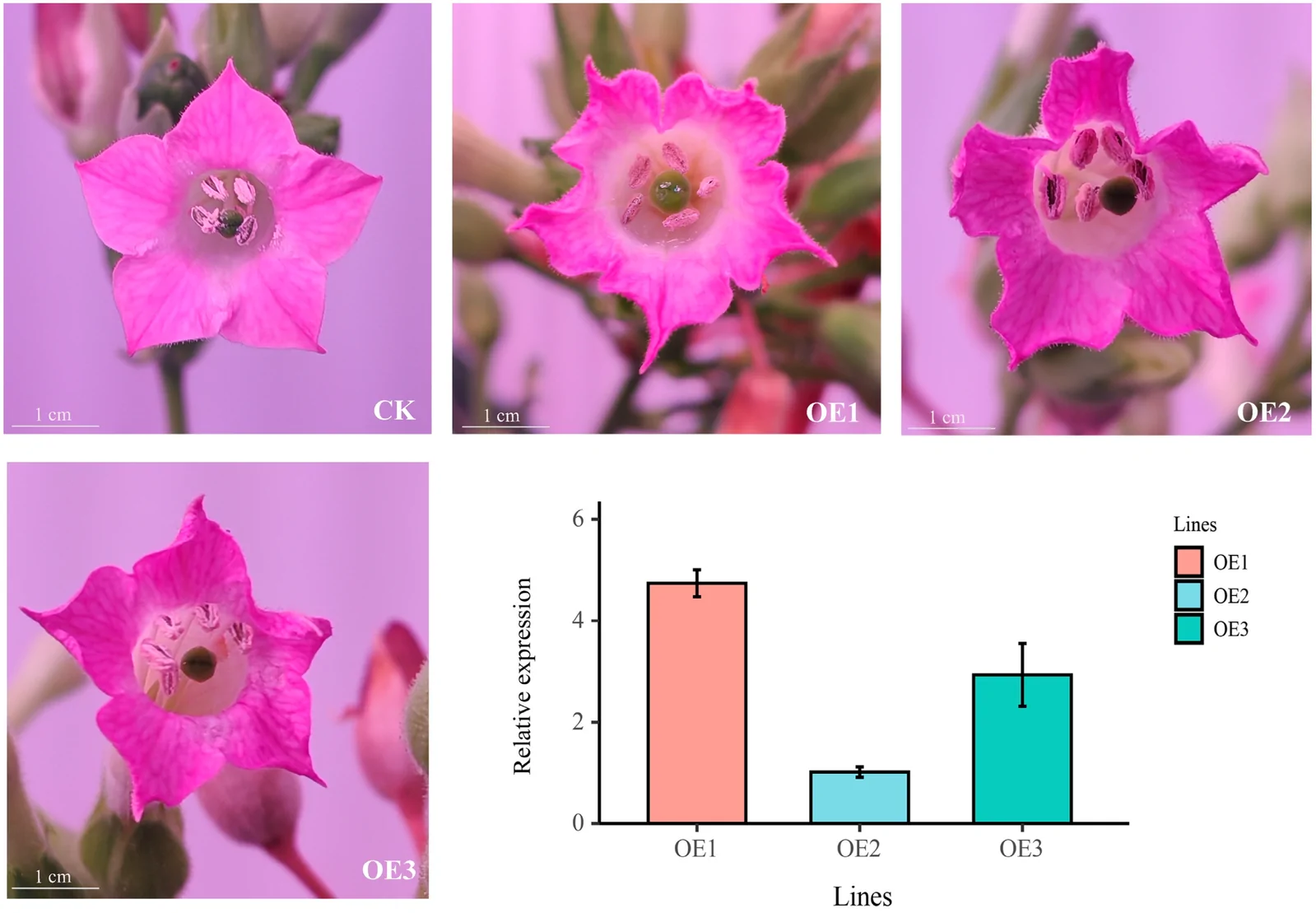
Phenotypic observations of *LsAP2/ERF10* OE lines in tobacco.

Heterologous overexpression of *LsAP2/ERF10* in tobacco disrupted the morphogenesis and anisotropic spatial growth of second-whorl floral organs. Compared with the normal corolla shape of control plants, the transgenic flower exhibited markedly acuminate corolla lobes with undulating and weakly reflexed margins, which reshaped the overall corolla into a stellate structure (Figure 9).

## 3. Discussion

The AP2/ERF transcription factors exhibit a wide range of functions, participating in the regulation of plant growth, stress responses, and biotic signaling, with functional specificity among different members [29]. *L. speciosa* is a typical woody ornamental that flowers in summer, bearing large and colorful blossoms and exhibiting considerable stress tolerance. With the availability of the *L. speciosa* genome, this study systematically identified and characterized the AP2/ERF transcription factor family in *L. speciosa*. A total of 248 AP2/ERF members were identified, and their evolutionary relationships, conserved motifs, cis-regulatory elements, and expression profiles of single- and double-flowered materials were analyzed. In addition, functional characterization of *LsAP2/ERF10* through ectopic expression in tobacco suggested its potential involvement in floral organ development. These analyses provided a fundamental basis for further elucidating the regulatory mechanisms of AP2/ERF genes in floral development in *L. speciosa*. However, the direct molecular mechanisms and regulatory relationships among AP2/ERF genes, miR172, and floral identity genes remain to be experimentally validated.

Phylogenetic analysis revealed that the AP2/ERF superfamily comprises five groups, with the ERF subfamily having the largest number of genes. Members of this subfamily are reported to play crucial roles in plant secondary metabolism. For instance, *ERF5* can transcriptionally activate the expression of *F3H*, thereby promoting anthocyanin accumulation in mulberry fruits [30]. In citrus, *ERF32*, *ERF33* and *RAV1* form a complex that significantly enhances the expression of chalcone isomerase gene *CHIL1*, thereby regulating flavonoid biosynthesis [31]. These findings suggest that ERF subfamily members in *L. speciosa* may similarly function in metabolic adaptation to environmental changes. The DREB subfamily, which has the second-largest number of genes in *L. speciosa*, specifically recognizes and binds to dehydration-responsive elements to respond to various stresses such as drought, low temperature, and high temperature, indicating its primary role in abiotic stress adaptation [32,33]. A total of 32 members were identified in the AP2 subfamily. In other species, the euAP2 group within this subfamily typically contains miR172 target sites and is involved in floral organ identity determination [18,34]. Based on the conserved role of the miR172-AP2 module in floral development, the euAP2 members identified in *L. speciosa* may potentially be associated with miR172-mediated regulation. However, this possibility remains hypothetical because miR172 target sites were not analyzed and experimentally validated in the present study. Future studies involving miR172 binding-site prediction, degradome analysis, and functional validation will be required to confirm this regulatory relationship.

The cis-acting elements in promoter regions play a pivotal role in regulating gene expression [35]. The *LsAP2/ERF* genes are enriched in cis-regulatory elements responsive to light, multiple phytohormones, and abiotic stresses, suggesting that this family functions as a key hub integrating diverse signaling pathways rather than acting as isolated regulators. Light-responsive elements were widely detected in the promoters of almost all family members, implying that this family may be extensively involved in plant growth and development through light signaling pathways. The enrichment of multiple hormone-responsive elements, particularly those responsive to ethylene and ABA, is consistent with the known roles of the ERF and DREB subfamilies as core components of ethylene and ABA signaling cascades [36,37]. Furthermore, the presence of tissue-specific elements, such as the meristem-associated CAT-box and palisade mesophyll differentiation-related elements, further suggests that certain *LsAP2/ERF* members may play regulatory roles in specific organ differentiation. Despite functional divergence among subfamilies, each subfamily harbors at least one ortholog shared between *A. thaliana* and *L. speciosa*, reflecting evolutionarily conserved relationships. Different subgroups of the *AP2/ERF* superfamily have undergone certain expansions or contractions during the evolution of various species [38]. Phylogenetically related *LsAP2/ERFs* share similar gene structures and conserved motifs, demonstrating high evolutionary conservation across species. However, conserved motifs vary among subfamilies: AP2 members typically contain two AP2 domains, while RAV members possess both AP2 and B3 domains, serving as key regulators of plant stress responses.

The AP2/ERF family members are unevenly distributed across all 24 chromosomes, with no apparent correlation with chromosome length. During evolution, plant genomes have undergone processes such as duplication and polyploidization, which can generate novel genes or functions through distinct mechanisms [39,40]. Gene duplication events are the primary driver of gene family expansion. We identified eight tandem duplication events and 184 segmental duplication events among *LsAP2/ERF* genes. These results suggest that segmental duplication played a pivotal role in the expansion of the *L. speciosa* AP2/ERF family. The collinearity results among different species indicate that *L. speciosa* shares a high degree of collinearity with other plants in the same genus, suggesting that the AP2/ERF families of different plants may have evolved from a common ancestor. STRING-based analysis predicted potential associations among *AP2/ERF* subfamily proteins, suggesting that different subfamilies may participate in shared regulatory pathways despite functional divergence. Although STRING analysis provides useful clues regarding potential functional associations among LsAP2/ERF proteins, these predicted interactions should not be considered experimentally confirmed protein–protein interactions. Future studies using yeast two-hybrid, bimolecular fluorescence complementation (BiFC), or co-immunoprecipitation assays are required to validate these relationships.

Many plant species are cultivated worldwide for their aesthetic characteristics, among which double flowers with many extra petals are particularly prized as an important ornamental trait due to their captivating appearance, which increases their commercial value. Recent studies have increasingly demonstrated that the expression of the class A gene AP2 is closely associated with the formation of double flowers [41,42]. AP2 transcription factors coordinate with other floral meristem identity genes to regulate organ development. In *A. thaliana* weak *ap2* mutants, reduced AP2 expression in the first and second whorls of floral organs correlates with sustained upregulation of *AGAMOUS* (*AG*) gene expression, leading to the transformation of sepals into carpels and a reduction in petal number. In strong *AP2* mutants, more pronounced *AG* expression in these whorls results in the complete absence of petals [43]. Previous studies have established that transcription factors contribute to double-flower formation in *L. speciosa* [26]. In this study, transcriptome sequencing combined with RT-qPCR validation revealed that expression level of *LsAP2/ERF* genes shows marked spatiotemporal specificity. Most *LsAP2/ERFs* were expressed during flower bud development, suggesting that they may participate in this process. In *L. speciosa*, AP2 subfamily members, as core genes in the regulatory network, are predominantly expressed in single-petal flower buds: *LsAP2/ERF9* and *LsAP2/ERF12* showed peak expression during early stages, while *LsAP2/ERF1*, *LsAP2/ERF13* and *LsAP2/ERF4* were mainly expressed in late stages. *LsAP2/ERF10*, *LsAP2/ERF2* and *LsAP2/ERF219* were constitutively expressed throughout all three flower bud stages, demonstrating stage-specific expression patterns. This expression pattern suggests that *AP2* genes may contribute to maintaining normal floral organ identity during early flower development. Reduced *AP2* expression in double-flowered materials may be associated with altered regulatory networks underlying stamen petaloidy and increased petal formation. However, whether *AP2* downregulation acts as a causal factor driving double-flower formation or represents a secondary consequence of floral developmental changes remains unclear and requires further functional investigation.

In the functional validation of *LsAP2/ERF* genes, heterologous overexpression of *LsAP2/ERF10* in tobacco significantly disrupted the morphogenesis and spatial anisotropic growth of second-whorl floral organs, resulting in acuminate corolla lobes with undulating and reflexed margins, and shifting the overall corolla outline toward a stellate configuration. In *Arabidopsis*, AP2 functions to specify petal identity and regulate petal development, and mutations in *AtAP2* lead to alterations in floral organ morphology [44]. This phenotypic similarity suggests that *LsAP2/ERF10* possesses evolutionarily conserved functions, further highlighting the essential role of euAP2 genes in petal morphogenesis. Although heterologous overexpression in tobacco provided preliminary evidence supporting the potential role of *LsAP2/ERF10* in petal morphogenesis, its endogenous biological function in *L. speciosa* remains to be further validated through loss-of-function approaches, such as VIGS or CRISPR/Cas-mediated genome editing.

As both AP2/ERF family members and class-A factors in the ABC model, AP2 subfamily transcription factors promote floral meristem establishment, regulate floral developmental genes, control sepal and petal development, and interact with other floral genes. In some plant species, certain AP2 members have been reported to suppress flowering by inhibiting the key floral integrator *SUPPRESSOR OF OVEREXPRESSION OF CO 1* (*SOC1*), while their transcript abundance is negatively modulated by *miR172* [42]. In angiosperms, the miR172-AP2 module’s regulation of double-flower formation is evolutionarily conserved. MiR172 effectively suppresses *AP2* gene expression through specific or non-specific binding to mRNAs, thereby altering their interactions with class B and C floral organ identity genes [45]. In *P. mume*, a 49-bp deletion in the *PmAP2L* gene leads to double-flower phenotypes [46]. Previous studies also revealed that a deletion within the 3′ terminal region of *Prupe.6G242400* resulted in truncated mRNA expression, generating a potential TO3-type transcription factor that escapes post-transcriptional regulation by miR172 [47]. However, whether a similar regulatory mechanism exists in *L. speciosa* remains unclear. In the present study, AP2 subfamily members exhibited distinct expression patterns between single- and double-petal flower buds, and overexpression of *LsAP2/ERF10* altered petal morphology in tobacco. These results indicate that AP2-related transcriptional regulation may participate in floral organ development. Nevertheless, direct regulation involving miR172, AGAMOUS, and downstream floral identity genes requires further validation through molecular interaction assays and genetic approaches.

Collectively, this study not only provides a comprehensive characterization of the AP2/ERF gene family in *L. speciosa*, but also identifies *LsAP2/ERF10* as a promising candidate involved in petal morphogenesis. These results establish a valuable genetic resource for future studies on floral organ development and facilitate the molecular breeding of ornamental *Lagerstroemia* cultivars.

## 4. Materials and Methods

### 4.1. Identification and Physicochemical Characterization of AP2/ERF Gene Family Members

The genome and annotation file of *A. thaliana* (TAIR10.1, GCF_000001735.4) were downloaded from the NCBI database (https://www.ncbi.nlm.nih.gov). The genome and annotation file of *L. speciosa* have been deposited at Figshare (https://doi.org/10.6084/m9.figshare.26861248) [28]. The gene and protein sequences of the *AtAP2/ERF* gene family were downloaded from the TAIR database (https://www.arabidopsis.org/) and used to perform a BLASTp search with an E-value threshold set at 1 × 10^−5^. Comparative BLAST analysis was performed between the AP2/ERF protein sequences of *L. speciosa* and *A. thaliana*. The Hidden Markov Model (HMM) profile of the conserved AP2/ERF domain (Pfam ID: PF00847) was downloaded from the Pfam database (http://pfam.sanger.ac.uk). Candidate protein sequences containing the AP2 domain were identified using HMMER (v3.3.2, E-value < 1 × 10^−5^). Incomplete and redundant sequences from the preliminary screening were manually removed. Sequences lacking the characteristic AP2/ERF domain were excluded, resulting in the final identification of 248 *LsAP2/ERF* genes. The physicochemical properties of the encoded proteins were predicted using the ExPASy ProtParam tool (http://web.ExPASy.org/protparam/, accessed on 19 March 2025).

### 4.2. Sequence Alignment and Phylogenetic Analysis

Multiple sequence alignment of the predicted LsAP2/ERF proteins was performed using ClustalW implemented in MEGA7 (v7.0.26) with default parameters, including a gap opening penalty of 10 and a gap extension penalty of 0.2. A phylogenetic tree was constructed using the neighbor-joining (NJ) method with the Poisson model, pairwise deletion for gaps and missing data, and 1000 bootstrap replicates to evaluate branch support. The LsAP2/ERF proteins were classified into subfamilies based on their phylogenetic relationships with corresponding AtAP2/ERF subgroups. The phylogenetic tree was refined and visualized using Figtree v1.4.4 for enhanced presentation.

### 4.3. Analysis of Cis-Acting Elements in the Promoters of AP2/ERF Genes

A 2000 bp upstream sequence of each identified *LsAP2/ERF* gene was extracted from the *L. speciosa* genome. These promoter sequences were subsequently analyzed for cis-acting regulatory elements using the online tool PlantCARE (https://bioinformatics.psb.ugent.be/webtools/plantcare/html/, accessed on 2 April 2025) and visualized using TBtools software (v2.323) [48].

### 4.4. Chromosomal Locations, Gene Duplication and Collinearity Analysis

The chromosomal lengths and positional information of identified *LsAP2/ERF* genes were extracted from genome annotation files. These genomic coordinates were subsequently used to generate a comprehensive chromosomal distribution map employing TBtools-II software. MCScanX (v1.0.0) with default parameters was used to determine the duplication events of *LsAP2/ERF* genes. The syntenic maps between *L. speciosa* and other species were constructed using the Dual Synteny Plotter of TBtools-II software (v2.323). The genome resource of *L. indica* was obtained from the National Genomics Data Centre under BioProject ID PRJCA013427. *L. suprareticulata* genomic data are not publicly available.

### 4.5. Analysis of Gene Expression and the Construction of Co-Expression Networks

The RNA-seq data used in this study were originally generated as de novo transcriptome sequencing before the availability of the reference genome (https://www.ncbi.nlm.nih.gov/sra/SRP176400, accessed on 7 January 2025). The raw reads were processed with fastp to remove adapters, reads with high N content, poly-A reads, and low-quality reads (Q ≤ 20 > 50%). rRNA contamination was eliminated using bowtie2 against a ribosomal database. Clean reads were aligned to the *L. speciosa* reference genome using HISAT2 (v2.1.0) with default parameters. Transcripts were assembled using Stringtie, and gene-level quantification was performed with RSEM to yield raw read counts and TPM values, the latter normalized by sequencing depth and gene length for down-stream analyses. This enabled comprehensive analysis of *LsAP2/ERFs* expression patterns across various floral developmental phases. Expression patterns were further visualized through heatmaps generated using TBtools software. The interaction network was inferred from the orthologs of *LsAP2/ERF* proteins using the STRING database (version 12.0, https://string-db.org/) (Table S4), and the co-expression network result was visualized using Cytoscape (v3.10.2).

### 4.6. Plant Materials, RNA Extraction and RT-qPCR Analysis

Single- and double-flower plants of *L. speciosa* were collected from the Guangxi Forestry Research Institute, Nanning, China (22°92′ N, 108°36′ E). Floral buds at different developmental stages were collected and designated as S1–S6 and D1–D6 for single- and double-flower phenotypes, respectively (Figure S1). Based on early morphological profiling that indicated that stages S1–S3 and D1–D3 represent the critical period governing floral organ differentiation and identity determination [26], flower buds from these three stages were selected for RNA extraction, with three biological replicates prepared for each stage. The flower buds were immediately frozen with liquid nitrogen and stored at −80 °C. The total RNA was extracted using a Total RNA kit (Aidlab Biotechnology, Beijing, China) and the first strand cDNA was synthesized using SuperScript™ IV VILO™ Master Mix (Thermo Fisher, Shanghai, China) according to the manufacturers’ instructions. RNA concentration and purity were measured using a NanoDrop spectrophotometer, and RNA integrity was evaluated by agarose gel electrophoresis. The RT-qPCR was performed using Taq Pro Universal SYBR qPCR Master Mix (Vazyme, Nanjing, China) on Applied Biosystems 7500 Real-Time PCR System. Each reaction was conducted in a 15 μL reaction volume comprising 7.5 μL of 2× Taq Pro Universal SYBR qPCR Master Mix, 0.5 μL of the forward primer, 0.5 μL of the reverse primer, 5.5 μL of ddH_2_O, and 1 μL of cDNA. The amplification conditions were set according to the manufacturer’s instructions. The 2^−ΔΔCT^ method was used to calculate the relative gene expression of *LsAP2/ERF* genes. All primer sequences are provided in Table S2; *Actin* was used as the internal reference gene [49].

### 4.7. Subcellular Localization

The coding sequences of selected LsAP2/ERF genes without stop codons were cloned into the pCAMBIA1300-GFP vector to generate a C-terminal GFP fusion construct under the control of the CaMV 35S promoter. The recombinant vectors were verified by PCR amplification and Sanger sequencing before transformation into Agrobacterium tumefaciens strain GV3101. For transient expression assays, Agrobacterium cultures were collected by centrifugation and resuspended in infiltration buffer containing MgCl_2_, MES and acetosyringone (AS) to induce virulence gene expression; the OD_600_ was adjusted to 0.6–0.8, and the samples were incubated in the dark at 28 °C for 4 h after resuspension. The suspension was infiltrated into the abaxial side of leaves from 4- to 5-leaf stage *Nicotiana benthamiana* plants. Following 24 h of dark culture, plants were exposed to light for 48 h. Leaf sections (0.5 × 0.5 cm) from infiltrated areas were excised and imaged using a confocal laser scanning microscope (TCS SP8; Leica, Wetzlar, Germany). GFP fluorescence was detected using an excitation wavelength of 488 nm and an emission range of 500–550 nm. DAPI fluorescence was detected using excitation at 405 nm and emission was measured between 430 and 480 nm. Chlorophyll autofluorescence was detected using excitation at 633 nm with emission collected between 650 and 750 nm. Primers required for construction of GFP-labeled overexpression vectors of *AP2/ERFs* are provided in Table S2. The empty vector was used as the control.

### 4.8. Agrobacterium-Mediated Transformation of Tobacco

*Agrobacterium*-mediated genetic transformation was performed using leaf discs excised from aseptically grown *Nicotiana tabacum*. The explants were pre-cultured on medium (MS medium supplemented with 7 g/L agar and 30 g/L sucrose, pH 5.8) for 2 d. *Agrobacterium tumefaciens* strains harboring pSuper1300 empty vector and p1300super-*LsAP2/ERF10*-GFP were verified by colony PCR after activation, and resuspended in liquid MS to OD_600_ = 0.5. The pre-cultured leaf discs were immersed in the bacterial suspension for 10 min with gentle agitation, blotted dry on sterile filter paper, and transferred to co-cultivation medium (MS medium containing 0.1 mg/L NAA, 1 mg/L 6-BA, 30 g/L sucrose, and 7 g/L agar, pH 5.8) for 2–3 d of dark incubation. Subsequently, the infected explants were transferred to selection medium (the above formulation supplemented with 50 mg/L hygromycin and 200 mg/L timentin), which was refreshed every 15 d, to induce callus formation and shoot regeneration. Regenerated shoots were excised and transferred to rooting medium (half-strength MS medium supplemented with 30 g/L sucrose, 7 g/L agar, 50 mg/L hygromycin, and 200 mg/L timentin) for root induction. Finally, rooted plantlets were acclimatized, transplanted into soil, and cultivated to flowering for phenotypic observation. Transformed plants were grown in a greenhouse under a photoperiod of 16 h light/8 h dark at 25 ± 2 °C. The transformation experiment was performed three times independently, and the presence of the *LsAP2/ERF10* transgene in positive lines was verified by PCR. A total of ten independent transgenic tobacco lines were generated through Agrobacterium-mediated transformation. The expression level of *LsAP2/ERF10* was measured by RT-qPCR using cDNA from corolla tubes of transgenic tobacco, using *NtActin* as the internal control. Relative expression levels were calculated using the 2^−ΔΔCT^ method. Because the target gene was not expressed in wild-type plants, one transgenic line with a stable and moderate expression level was selected as the calibrator for comparative analyses among independent transgenic lines.

## 5. Conclusions

*Lagerstroemia speciosa* holds a distinctive position among summer-flowering plants. This study identified 248 *AP2/ERF* genes that were phylogenetically classified into five distinct subfamilies. These *LsAP2/ERF* genes displayed an uneven distribution across all 24 chromosomes, and segmental duplications served as the primary driver of family expansion. Cis-element analysis of *LsAP2/ERF* gene promoters identified multiple predicted cis-acting elements associated with light and phytohormone responses. Several AP2 subfamily members, including *LsAP2/ERF09* and *LsAP2/ERF10,* exhibited distinct spatiotemporal expression patterns during floral bud development and are predominantly localized to the nucleus. Furthermore, heterologous overexpression of *LsAP2/ERF10* in tobacco significantly altered petal morphology, supporting its potential role in petal development. Collectively, these findings provide a framework and mechanistic insights into AP2/ERF-mediated floral organ formation in *L. speciosa*.

## Data Availability

The original contributions presented in this study are included in the article/Supplementary Materials. Further inquiries can be directed to the corresponding authors.

## Supplementary Materials

The following supporting information can be downloaded at: https://www.mdpi.com/article/10.3390/plants15162427/s1, Table S1: The information for 248 *LsAP2/ERF* genes in *Lagerstroemia* species; Table S2: Primer sequences for *LsAP2/ERF* genes; Figure S1: The flower buds of *L. speciosa* at different developmental stages. S1–S6, six different development stages of single-petaled flowers (S); D1–D6, six different development stages of double-petaled flowers (D). Table S3: All 147 AtAP2/ERF protein sequences from *Arabidopsis thaliana*. Table S4: Information data derived from STRING-based interaction prediction.
