# Supplementary material for: Genome-Wide Superfamily Profiling Reveals Key Regulator LsAP2/ERF10 That Governs Petal Morphology in Lagerstroemia speciosa
Source: Plants (Basel). 2026 Aug 9;15(16):2427. doi: 10.3390/plants15162427 (PMC13516282; doi:10.3390/plants15162427)
Supplement: Supplementary file 1 [file plants-15-02427-s001.zip › Figure S1.pdf]

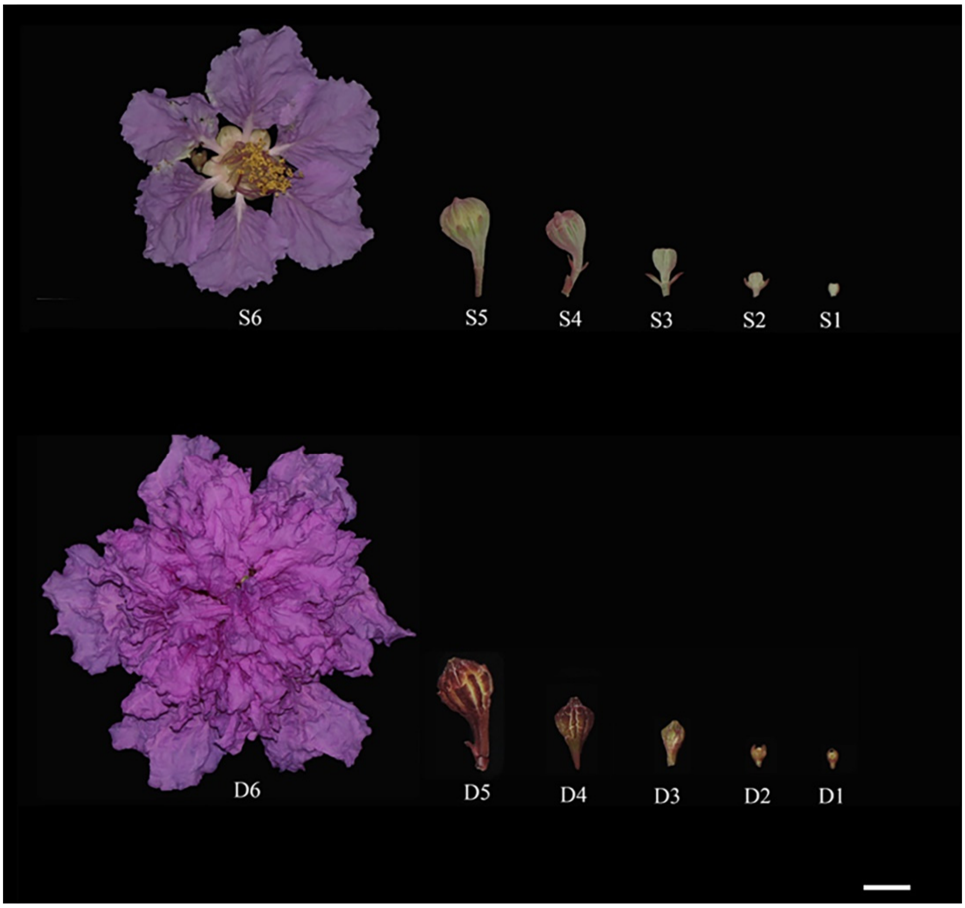

Figure S1. The flower buds of *L. speciosa* at different developmental stages. S1-S6, six different development stages of single-petaled flowers(S); D1-D6, six different development stages of double-petaled flowers(D).
